# Ultrasonic-Assisted Extrusion Processing for Enhancing Physical Properties of High-Density Polyethylene by Flow-Induced Crystallization

**DOI:** 10.1021/acsapm.5c03508

**Published:** 2026-01-06

**Authors:** Mansoureh Jamalzadeh, David O. Kazmer, Patrick Casey, E. Bryan Coughlin, Margaret J. Sobkowicz

**Affiliations:** † Plastics Engineering Department, 10833 University of Massachusetts Lowell, Lowell, Massachusetts 01854, United States; ‡ Polymer Science and Engineering Department, 14707 University of Massachusetts Amherst, Amherst, Massachusetts 01004, United States

**Keywords:** flow-induced crystallization, ultrasonic-assisted extrusion processing, oxygen permeation, film extrusion, design for recycling

## Abstract

The evolution of crystallinity resulting from stress imposed on a melt, known as flow-induced crystallinity, can strongly influence the mechanical and physical properties of semicrystalline polymers. This study investigates shear-induced crystallization by applying an ultrasonic field to the melt flow as it passes through dies with various geometries. A custom-built sonication die is employed for controlling the dynamic temperature and shear environment, resulting in molecular alignment and potential for flow-induced crystallization. Application of both conventional and ultrasonic shear rates at the equilibrium melt temperature of high-density polyethylene (HDPE) was investigated to accelerate crystallinity and manipulate the crystal morphology across the film in pursuit of improved mechanical and gas barrier properties without the need for additives or other polymer layers. The relationships among ultrasonic-assisted extrusion processing, polymer structure, and performance were analyzed using wide- and small-angle X-ray scattering (WAXS and SAXS), tensile testing, and oxygen transmission rate (OTR) analysis. Multiple linear regression models were implemented to predict the correlation among HDPE structure, process, and properties. Structural analysis revealed that both conventional and ultrasonic shear rates had the most significant influence on lamellar spacing and redistribution of rigid and soft amorphous fractions within the crystalline domains, ultimately dictating the mechanical and physical properties of the films. The goal is to explore the potential of the ultrasonic-assisted high crystallinity monolayer that can replace some of the functionality of complex, heterogeneous multilayer packaging with a single-material film having enhanced oxygen barrier properties.

## Graphical abstract

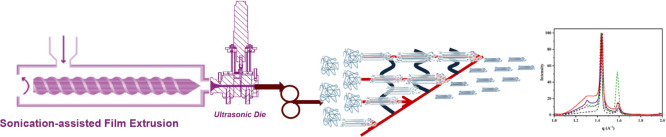

## Introduction

Polyethylene films are ubiquitous due to their low cost and ease of processing; however, their relatively high oxygen permeability can negatively impact the shelf life of oxygen-sensitive products. The oxygen transmission rate (OTR) for HDPE typically ranges from 400 to 6000 cc·mil/m^2^·day, influenced by the crystalline structure.^1^ To overcome the high oxygen permeability of polyethylene films, many plastic films are composed of multiple polymer layers and other heterogeneous materials such as high barrier resin. This approach achieves a low OTR of less than 100 cc·mil/m^2^·day.^2^ Nevertheless, the heterogeneous multilayered films are not recyclable. The enhancement of oxygen barrier properties in monopolymer films is significantly influenced by the crystal structure.^3^ For instance, the physical size and distribution of crystallites, particularly the formation of large crystals oriented in the plane of the film, can significantly reduce oxygen permeability.^4^ This improvement arises from increased tortuosity and reduced free volume within the amorphous regions between the larger crystals. Additionally, the polymer’s crystal structure and morphology are determined during continuous processing under complex conditions that typically involve high cooling rates, shear stress, and pressure.^5,6^ Researchers have explored these factors in various studies related to nonisothermal crystallization, flow-induced crystallization (FIC), and polymer crystallization under pressure.^7,8^ The Avrami equation serves as a foundational model for characterizing isothermal crystallization kinetics, providing a theoretical framework for understanding the nucleation and growth processes. To enhance the accuracy of process modeling, integrating both isothermal and nonisothermal experimental data is crucial. Alternative approaches, such as the Ozawa models, have been developed to apply nonisothermal predictions.^9,10^ The crystallization kinetics of high-density polyethylene under nonisothermal conditions starts with random nucleation followed by isotropic growth of spherulites.^11^

Complementary to the quiescent crystallization kinetics models are crystallization processes measured while the polymer is under deformation.^12,13^ When deformation is applied to the polymer melt at or near the crystallization temperature just before or during quenching, FIC can significantly enhance the concentration of nuclei and even change the final crystalline morphology and anisotropy.^14^ The resulting crystalline morphology is influenced by the specific work (eq 1), which is defined by the processing parameters; here σ is the stress, γ is the strain/deformation, μ is the viscosity at the applied deformation rate, and *t* is the duration of the applied stress.^14^

1 $$W=\sigma \gamma =\mu {\mathrm{\gamma ̇}}^{2}t$$

The minimum shear rate required for FIC can be determined by the Rouse relaxation time (τ_R_) and reptation relaxation time (τ_d_) of the polymer chain (eq 2).

2 γ̇=1τR⁣τR=(M/Me)2τe⁣τd=3MMeτR

*M*_e_ and τ_e_ are the molecular weight and Rouse relaxation time of a polymer chain at the entanglement molecular weight, respectively. As the shear rate increases above the minimum inverse τ_R_ of the longest chains in the molecular weight distribution, more nuclei form leading to an increased crystallization rate.^15^

Some works have investigated FIC during injection molding, but it is beneficial to be able to apply a tunable stress to control FIC at flow rates applicable to various melt processing operations.^16^ The shear stress required to align molecules and achieve FIC can be applied by introducing an external field such as ultrasonic energy during the extrusion process. We propose that ultrasonic-assisted crystallization has the potential to both accelerate the crystallization process and alter the crystal structure, without requiring specialized polymer formulations or additives and without causing the detrimental effects often associated with other forms of electromagnetic radiation on polymers.^17^ This approach could enhance the mechanical and barrier properties of polyethylene films, making it a versatile solution for producing high-performance, inherently recyclable films.^18^ Ultrasound-assisted crystallization (called sono-nucleation) has previously been shown to accelerate the nucleation rate and better control the formation and growth of crystals in small organic molecules and slow crystallizing polymers.^19,20^ However, the limited understanding of how the ultrasonic field interacts with the polymer melt restricts its practical application during polymer processing. Therefore, it is crucial to examine the effect of factors such as polymer structure, processing conditions, and ultrasonic power in improving crystallinity.^21^

The use of ultrasonic fields to enhance polymer melt processing was demonstrated in the pioneering work of Isayev et al. They introduced ultrasound-coupled extrusion for the devulcanization of vulcanized elastomers. Their work showed that ultrasound waves can effectively break the C–S and S–S bonds of cross-linked rubber, leading to devulcanization of the rubber.^22^ Devulcanized rubber obtained through ultrasonic extrusion resulted in a smaller gel fraction, making it suitable for the production of new rubber products or compounding with virgin rubbers.^23^ Furthermore, molecular weight analysis of ultrasonic treatment of rubber revealed simultaneous reactions involving gel formation, branching, and degradation.^24^ The study of ultrasonic irradiation of polypropylene (PP) revealed that the degradation of PP increases with the sonication time. The authors found that the degradation was higher for the 20 kHz frequency compared with other frequencies. Additionally, the molecular weight distribution of polypropylene widened as the irradiation time increased. Ultrasonic-assisted extrusion enhances nanoparticle dispersion in polymer melts without causing chemical reactions, unlike other electromagnetic methods.^25,26^ The use of the external field aided the formation of well-dispersed nanocomposites with high-density polyethylene (HDPE) and polypropylene (PP) due to intercalation/exfoliation of clay and polymer matrix degradation under the influence of ultrasound.^27,28^ The application of ultrasound during foam extrusion was shown to impact the foam density by reducing the size of cells and narrowing their size distribution. This effect was attributed to the disruption of large cells and the prevention of coalescence of small cells.^29^

Experimental studies have explored the interaction of polymers with sound waves during extrusion, revealing significant alterations in the material properties. These effects are influenced by parameters such as melt temperature, flow rate, and oscillation characteristics.^30^ The application of ultrasound at the die has been shown to reduce the die pressure and extrudate swelling. Additionally, the introduction of oscillatory energy could lead to a molecular weight reduction and decreased viscosity, indicating potential polymer degradation. The extent of these effects depends on factors such as frequency, amplitude, and flow rate.^31^ The dissipation of oscillatory energy and heat conduction also influence the polymer melt flow behavior. Increasing either the frequency or deformation amplitude at a constant flow rate reduces die pressure while raising the melt temperature due to viscous dissipation. Theoretical studies suggest that at lower frequencies, pressure reduction is primarily attributed to viscoelastic effects, whereas at higher frequencies, both increased temperature and viscoelasticity contribute to viscosity reduction.^31^ Depending on the geometry of the sonication area, pressure changes in the melt can also lead to compression and pulsing of the flow rate.

The ultrasound effect has been described as acoustic cavitation in Newtonian fluids, a process consisting of nucleation, bubble growth, and implosive collapse. The acoustic cavitation phenomenon is suppressed in non-Newtonian viscoelastic systems, and an alternative mechanism is that the ultrasound energy interacts with the vibrational motion and relaxation dynamics in polymers, which are intrinsically linked to the material’s viscoelastic properties and molecular characteristics. Increasing ultrasound vibration frequency and amplitude has been shown to reduce the viscosity of certain polymer melts due to the application of shear stress.^32^ This phenomenon offers potential advantages for processing high-molecular-weight polymers and highly filled composites by lowering energy consumption and improving melt homogeneity.^33^

This research investigated the impact of an ultrasonic field applied during polyethylene sheet extrusion on its crystallinity. The nonisothermal crystallinity kinetics of HDPE were analyzed by the Ozawa model. Subsequently, the susceptibility of the HDPE structure on flow-induced crystallinity was studied using a parallel plate rheometer by comparing the crystallization induction time in the quiescent state with that achieved by applying a preshear before quenching. The effects of processing variables including flow rate, cooling rate, and draw ratio during cast film extrusion as well as shear rate and sonication were evaluated on HDPE. An ultrasound field was applied to a die plate with different geometries just before quenching the polymer to manipulate crystallinity and crystal structure. Furthermore, the effects of the ultrasonic field and its potential to induce crystallinity (sonication-induced crystallization) were compared to those of shear-induced crystallization via conventional melt flow. The crystal structure of HDPE after processing was characterized by using X-ray diffraction (XRD) techniques, including WAXS and SAXS, in conjunction with measurements of mechanical and physical properties. Finally, the correlation of structure, process, and properties of HDPE was analyzed by implementing a multiple linear regression model.

## Experimental Approach

### Materials

The experiment was conducted using high-density polyethylene (HDPE) provided by ExxonMobil. This HDPE has a density of 0.963 g/cm^3^ and an MFI of 0.78 g/10 min. The MFI data for the resin were reported at 190 °C with a load of 2.16 kg. The molecular weight was determined using high-temperature gas chromatography (HT-GPC), resulting in a *M*_n_ of 126.5 kg/mol and a *M*_w_ of 193 kg/mol. The polydispersity index (PDI) was calculated to be 6.54.

### Rheological Protocol

The rheological properties of the samples were measured using a parallel plate rheometer (ARES-G2) equipped with 25 mm stainless steel plates from TA Instruments (New Castle, DE). The disk samples were prepared using a microinjection molding unit from Xplore Instruments (Sittard, The Netherlands). To obtain a master curve for HDPE, we initially performed a strain sweep to determine the linear viscoelastic region. A strain value of 10% was selected for subsequent frequency sweep tests. Frequency sweep tests were conducted over an angular frequency range of 0.1 to 100 rad/s across temperatures ranging from 140 to 350 °C with a 50 °C increment under nitrogen gas. The frequency sweep data at various temperatures were shifted to a higher frequency range in the temperature range of 140 °C by applying the time–temperature superposition method using TRIOS software to calculate the temperature shift factor.

A dynamic crystallization study was undertaken to further probe the susceptibility of HDPE to FIC using the parallel plate rheometer with the following procedure. In this rheology protocol, samples were initially heated to 180 °C and soaked for 3 min (step 1) to eliminate any crystalline structure. Next, the samples were cooled from 180 °C to a shearing temperature of 140 °C at a rate of 20 °C/min (step 2). Subsequently, a stress growth experiment was conducted by applying a steady shear rate in the range of 0 to 3 s^–1^ over 100 s. At zero shear, the sample was in a quiescent state, and at higher value, a preshear was applied for 100 s. After the shearing step 3, the sample was quenched from 140 °C to its crystallization temperature (*T*_c_) at a rate of 5 °C/min (step 4). Finally, an oscillation time sweep was performed at a strain amplitude of 1% and an angular frequency of 1 rad/s for 1000 s, and the growth in viscoelastic response of HDPE was monitored as crystallization began (step 5). The procedure schematic for this study is shown in the Supporting Information (Figure S1).

### Cast Film Extrusion Processing

The cast film extrusion process was executed using a single screw extruder from Collin Lab & Pilot Solutions, complemented by a takeoff unit from Thermo Haake equipped with three driven rollers cooled using a constant temperature circulating bath. As depicted in Figure 1, an extrusion die was custom designed and fabricated accompanied by an ultrasonic generator (model LC20-2000, Ligke Ultrasonics). This ultrasonic generator provides a continuous and adjustable power output of up to 2000 W at a frequency of 20 kHz. Additionally, a set of sonication plates was designed and manufactured by SendCutSend (Reno, NV) from 7075-T6 aluminum. Three sonication plates (rectangular opening, anvil, and Hole pattern) with a 3.2 mm thickness used in this research are illustrated with detailed design in Figure 1. The melt flow passes through a rectangular opening plate with dimensions of 33 mm (length) and 4.33 mm (height). The hole pattern plate features holes drilled with a diameter of 1 mm. These sonication plates effectively transfer the maximum sonication energy to the melt, facilitating the shearing of the melt flow by providing 12.5 μm amplitude at a frequency of 20 kHz. Although the ultrasonic-induced strain amplitude is small, the frequency of 20 kHz can cumulatively enhance chain alignment and nucleation, promoting crystallinity similarly to a flow-induced crystallization process.

**Figure 1:**
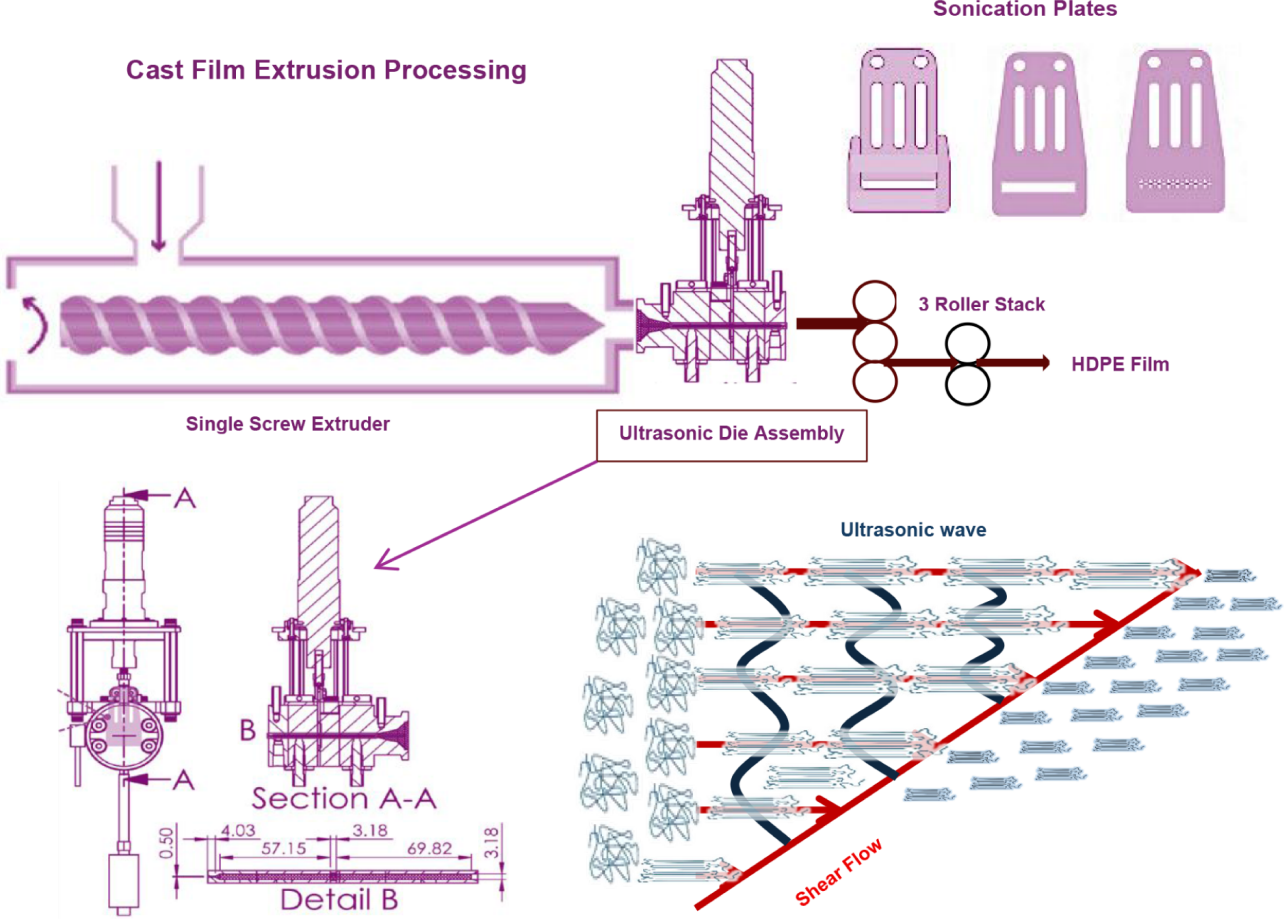
Ultrasonic-assisted extrusion processing schematic with different plates (anvil, rectangular opening (blank), and hole pattern) and crystal structure evolution under both shear flow and ultrasonic field.

During the extrusion process, the temperature profile was set to surpass the melting point of HDPE, reaching a maximum of 180 °C. Subsequently, it gradually decreased to 145 °C at the die inlet before the ultrasonic plate. The temperature at the die outlet after the sonication plate was set to 140 °C, and this temperature gradient was used with the intent of applying shear stress at a temperature around the equilibrium melt temperature of HDPE. It was demonstrated that FIC is effective when preshear is applied at the equilibrium melt temperature of the polymer chain.^15^ At this temperature, polymer chains are sufficiently mobile to be stretched under shear while still being close to the crystallization regime. According to nonlinear Hoffman–Weeks extrapolation, the equilibrium melt temperature of an infinitely long chain polyethylene chain is reported to be 141.4 ± 0.8 °C.^34^ We hypothesize that the ultrasonic field will have more influence on the crystallization process at the equilibrium melt temperature. Then, the melt flow exiting the die was cooled under stretching on the roll stack to form a film.

The combined effects of melt flow rate, draw ratio, cooling rate, and shear rate, as well as ultrasonic amplitude on the crystal structure during cast film extrusion were studied through a design of experiments (DOE) to assess the impact of each variable independently (Table 1). The effect of the cooling rate was investigated by varying the temperature of the roll stack. The temperatures of the roll stack were selected to be 120 and 60 °C, corresponding to the onset and end of crystallization peak temperatures, respectively, as indicated by the DSC results presented in the Supporting Information (Figure S2).^35^ Additionally, the effect of supercooling was also investigated by setting the roll stack temperature to a third setting of 25 °C.

**Table 1:** Processing Parameters Per Each Run of One Factor at a Time (OFAT) Design of Experiments (DOE)

Run #	Investigated Processing Variable (Label)	Screw speed (rpm)	Draw ratio	***T***_Roll_ (°C)	Plate Pattern
0	Center point (F15, D5, T60)	15	5	60	Rectangular Opening/Holes
1	Low flow rate (F10)	10	5	60	Rectangular Opening/Holes
2	High flow rate (F20)	20	5	60	Rectangular Opening/Holes
3	Low draw ratio (D2.5)	15	2.5	60	Rectangular Opening/Holes
4	High draw ratio (D10)	15	10	60	Rectangular Opening/Holes
5	Low roll temperature (T20)	15	5	20	Rectangular Opening/Holes
6	High roll temperature (T120)	15	5	120	Rectangular Opening/Holes

The impact of the draw ratio on the HDPE crystal structure was also tested by varying the puller speeds. The linear speed of the puller was adjusted based on the desired draw ratios of 2.5, 5, and 10, which could be achieved by determining the linear speed of the melt flow at the outlet of the die. In this way, a volumetric flow rate was calculated during a specific time, and the linear speed was calculated according to the cross-sectional area of the die at the outlet. The calculated linear velocity did not consider densification during solidification. The film with a draw ratio of 5 had a thickness of approximately 300 ± 20 μm.

The effect of the flow rate was examined by running the extruder at different screw speeds while adjusting the puller speed to get a constant film thickness. The shear rate at the sonication die was altered by passing the melt flow through either the rectangular opening plate or the small holes (Figure 1). The shear rate at the plate-melt interfaces was calculated using eq 3 for the sonication plate with holes and eq 4 for the sonication plate with a rectangular opening pattern, considering the volumetric flow rate that passes through the cross-section of the corresponding flow channel(s).

3 $$\mathrm{\gamma ̇}=\frac{4Q}{\pi r^{3}}$$

4 $$\mathrm{\gamma ̇}=\frac{6Q}{wh^{2}}$$

Here, *Q* is volumetric melt flow rate across die, *r* is the radius of the holes, and *w* and *h* are the width and thickness of the rectangular opening, respectively. Table 1 presents the extrusion parameters employed during the DOE study conducted for this research. In addition to the rectangular opening plate and the plate with holes, the anvil design was used to induce volumetric changes as the melt flow passed through the same rectangular opening channel and was subjected to ultrasonic waves. Without ultrasound on, the anvil design is identical to the rectangular opening plate. This design is anticipated to transfer the ultrasonic field with higher power, resulting in a higher influence on the melt flow compared to the rectangular opening plate.

The estimated shear rates with different plates, including the implementation of the ultrasonic field (displacement of 12.5 μm amplitude at a frequency of 20 kHz), are listed in Table 2.

**Table 2:** Estimated Shear Rate under Each Processing Condition

Processing variable	Screw speed (rpm)	Draw ratio	***T***_Roll_ (°C)	Plate pattern	Ultrasonic amplitude (μm)	Shear rate γ̇ (1/s)
CenterPoint F15, D5, T60	15	5	60	Rectangular	0	2
				Rectangular	12.5	100
				Anvil	12.5	200
				Holes	0	100
				Holes	12.5	1000

### Differential Scanning Calorimetry

Differential scanning calorimetry (DSC) was performed using DSC 3+ by METTLER TOLEDO. The heat flow enthalpy was measured in a heat–cool–heat cycle within 25 to 200 °C and a rate of ± 10 °C/min. The DSC test was conducted at different cooling rates from 10 to 30 °C/min in 5 °C increments. This was performed to analyze the nonisothermal crystallization kinetics of HDPE by applying the Ozawa model per each cooling rate.

### X-ray Scattering

X-ray scattering experiments were done using a Rigaku SmartLab, equipped with a copper X-ray tube (λ = 0.154 nm) and a 2D HyPix 3000 detector. Data acquisition was conducted using a current of 2 mA and a voltage of 20 kV for a duration of 10 min. Samples were measured in transmission geometry with 2D image plate detection. The film was placed in the beam, with the machine stretching direction oriented vertically. The raw intensity data were analyzed with the SmartLab analysis software by locating the scattering peak position using the second derivative method (with a sigma cut of 3 for noise filtering) and fitting the corrected data with a split pseudoVoigt model.

### Small-Angle X-ray Scattering

The SAXS method was employed to determine the dominant lamellar spacing (*d*), which represents the folding period of the polyethylene crystals. The peak value in the scattering intensity versus scattering vector (*q*) was used to calculate *d*, as per eq 5

5 d=2πq

### Wide-Angle X-ray Scattering

The WAXS pattern was used to examine the crystalline structure, including crystallinity percentage, crystal size, and orientation degree, presented in the Supporting Information as Figure S5. The orthorhombic crystal structure of HDPE was characterized by two prominent X-ray diffraction reflections at 2θ ∼ 21° and 23°, corresponding to the (110) and (200) crystallographic planes, respectively.^36^ The crystalline domain size (*D*) was estimated using Scherrer’s formula, eq 6, which shows an inverse proportionality between *D* and the full-width half-maximum (fwhm) of the scattered X-ray peak represented by *B*_hkl_ (2θ). The wavelength of X-rays, *λ*, is 1.54 Å for CuKα radiation.

6 D=0.94λBhkl⁡cos⁡(θ)

The crystallinity percentage was calculated based on the intensity integration of two sharp crystal peaks (110 and 200 planes) divided by the total crystal peaks and amorphous halo of the scattered X-rays.

The degree of crystal orientation was measured quantitatively by using the WAXS scattering intensity pattern depicted in Figure 2. Variations in scattering intensity appeared throughout the two-dimensional WAXS image, and the orientation index (defined by eq 7) was employed to assess the crystal’s anisotropy. This index was calculated as the ratio of the integrated scattering intensity across specific azimuthal angle segments (β = 140–240°), which relate to the 110 plane (at 2*q* ≈ 21.3°), to the average scattering intensity across the full azimuthal range (β = 0–360°).

7 Orientationdegree(%)=[Iq@β=140−240Iq@β=0−360]×100

**Figure 2:**
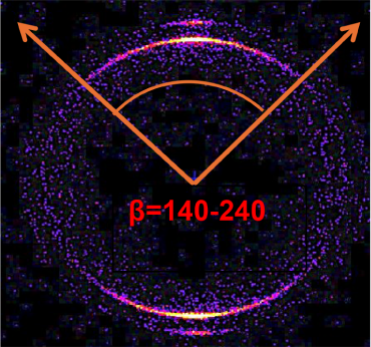
WAXS scattering pattern at β = 140°–240° for a sample at the central processing conditions (F15, D5, T60).

### High Temperature Gel Permeation Chromatography

Molecular weights and dispersity were measured by using high temperature gel permeation chromatography (HT-GPC) with infrared detection on a Polymer Char GPC-IR instrument. All samples were dissolved in 1,2,4-trichlorobenzene at 150 °C with a 90 min dissolution time. Measurement was conducted at a concentration of 1 mg/mL, and molecular weights were determined using a calibration curve derived from 16 narrow molecular weight polystyrene (PS) standards.

### Tensile Properties

The tensile properties were determined using an Instron model 5966 tensile testing machine following the ASTM D638 procedure at ambient temperature. The samples were cut into dog-bone-shaped specimens (IV) from the HDPE film. Five samples were taken per each processing variable of DOE, and the tensile testing was conducted at an extension rate of 10 mm/min, corresponding to a strain rate of 0.5%/s.

### Oxygen Transmission Rate

Oxygen transmission rate (OTR) was measured at ambient temperature and 0% humidity using a Mocon instrument (Ametek OX TRAN 2/22 series) in accordance with ASTM D3985. Foil masks with circular areas of 1 and 5 cm^2^ were employed to reduce the area of permeation on the HDPE films. OTR testing was repeated at least twice per sample to ensure no outlying behavior, and the mean result was reported.

## Results and Discussion

### Crystallization Kinetics

The nonisothermal crystallization kinetics of HDPE was studied by performing nonisothermal DSC tests at different cooling rates, then applying the Ozawa model (eqs 8 and 9) to extract kinetic parameters at different temperatures in which *X*_c_ is the fractional crystallinity, *k*(*T*) is the crystallization rate constant, *n* is the Ozawa exponent, and *Q* is the cooling rate.^37^ The temperature ranges were selected to span from the crystallization induction temperature to the crystallization temperature peak to ensure fidelity of the Ozawa model. Increasing the cooling rate led to a shift in the crystallization onset and peak to a lower temperature. This result indicated that supercooling was necessary to initiate crystallization and nucleation.^38^ The plot of ln­(−ln­(1 – *X*_c_)) versus ln(1Q) is depicted in Figure 3, and the linear fit was applied to determine *k*(*T*) and *n*.^39^ These fitted coefficients are presented in Table 3.

8 $$X_{c}=1-e^{-k\left(T\right){\frac{1}{Q}}^{n}}$$

9 ln(−ln(1−Xc))=ln(k(T))+n⁡ln(1Q)

**Figure 3:**
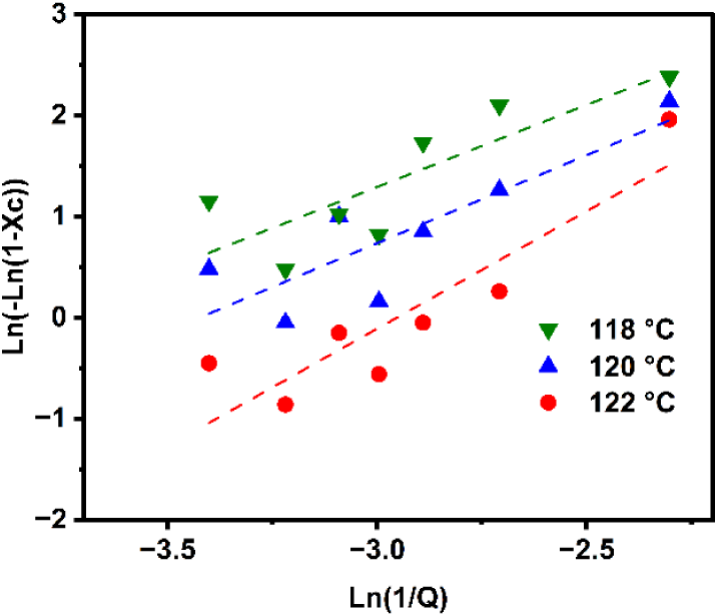
Ozawa model analysis.

**Table 3:** Ozawa Model Constants at Various Temperatures with the Fitted Model Coefficients

Temperature (°C)	*k*(*T*)	*n*	*R* ^2^ value
122	6.8	2.32	0.81
120	5.95	1.74	0.72
118	6.16	1.62	0.78

The temperature dependence of the growth and nucleation rate of HDPE crystallinity, expressed as *k*(*T*), exhibits no significant change in this range of temperature meaning that the crystallization rate is relatively stable in this range of cooling rate for HDPE. The exponent *n*, approximately 2 at the higher crystallization temperatures, is indicative of 2D lamellar growth.^40^ However, a reduction in *n* is observed as crystallization proceeds under deeper undercooling, indicating a transition toward more constrained crystallization kinetics. This reduction in *n* also captures the combined effects of reduced chain mobility, increased viscosity, and suppressed nucleation activity at lower temperatures. This behavior is consistent with overall crystallization rates governed largely by nucleation and strong radial growth of crystals.^41^

### Flow-Induced Crystallization

The crystallization kinetics of HDPE, as determined using the Ozawa model, demonstrates how the cooling rate and temperature influence the overall crystallization rate and morphology under steady-state conditions. However, in polymer processing, crystallization occurs under shear or elongational flow, which can substantially alter the nucleation and growth mechanisms by stretching and orienting the polymer chain. Stretching a polymer chain requires a shear rate higher than the inverse of the Rouse relaxation time for the longest chain.^15^ The relaxation time of a polymer chain also depends on its molecular weight. As shown in Figure S3 of the Supporting Information, the HDPE master curve at 140 °C exhibits a high zero-shear viscosity, which is attributed to its high molecular weight. Consequently, the onset of shear thinning behavior occurs at a lower frequency, indicating a long relaxation time and making it more sensitive to FIC. This aligns with the fact that a polymer chain with a higher molecular weight has a longer relaxation time due to the presence of more entanglements per chain.^42^ Stretching a long polymer chain during the process by reducing its entropy energy leads to a higher concentration of nuclei, resulting in faster crystallization with smaller crystal sizes at a given undercooling.

Accordingly, the effect of FIC on the crystallization kinetics of HDPE was studied by performing a rheology test with the procedure mentioned in the experimental approach. The isothermal crystallization induction time was identified at the crossover of loss and storage moduli (tan⁡δ=G″(ω0)G′(ω0)) , e.g., when tan δ = 1. This was chosen as indicative that the forming crystallites have increased the resistance to flow such that elasticity has begun to dominate over flow, as illustrated in Figure 4a. A range of temperatures was examined around the crystallization onset time to find the best temperature for monitoring the crystallization process. At high temperature, crystallization takes an inconveniently long time to begin, while at low temperature, crystallization is very fast and the induction time cannot be captured before completing the quench step. Due to this practical limitation, a temperature that yielded a crystallization induction time of less than 1000 s was chosen, and the effect of preshear at that temperature was examined. It was found that an appropriate temperature for crystallization induction was 128 °C. As shown in Figure 4b, applying preshear at 128 °C resulted in an earlier crystallization induction time. Figure 4c shows the crystallization induction time plotted vs preshear rate and further demonstrates that the selected HDPE is sensitive to the applied shear rate, as its crystallization time exhibits a significant decrease at a preshear rate of only 3 s^–1^, indicating shear-induced crystallization. This behavior can be attributed to the high molecular weight of the selected HDPE, which corresponds to a higher Rouse relaxation time, making it suitable for investigating FIC phenomena during cast film extrusion.

**Figure 4:**
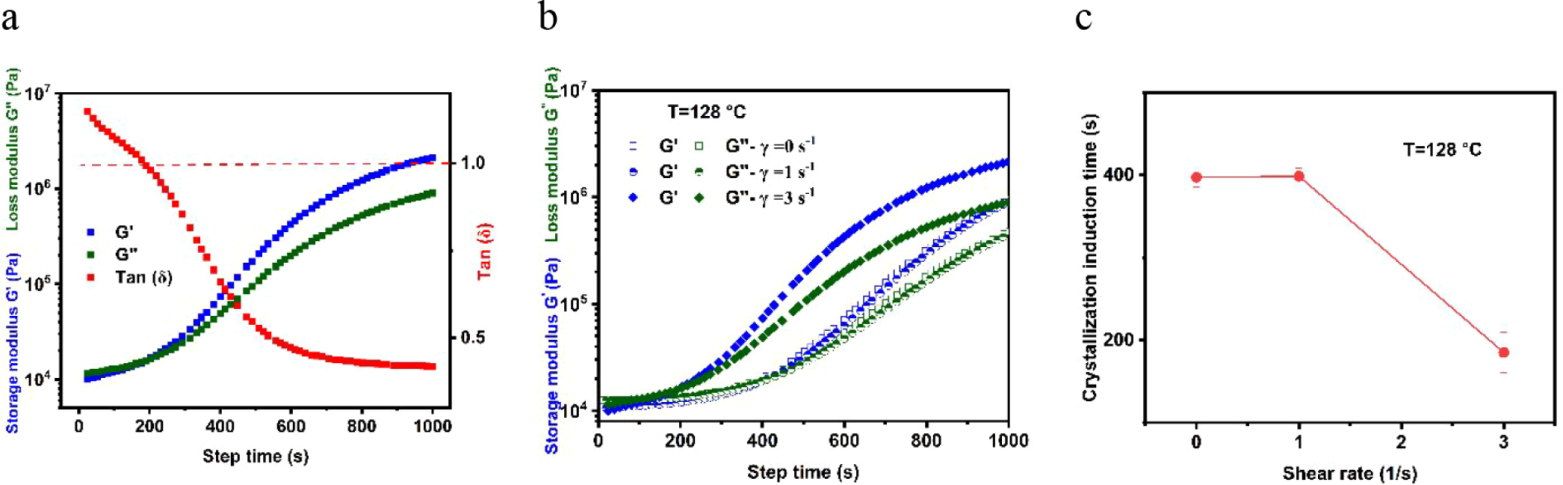
Oscillation time sweep test results for isothermal crystallization induction: (a) illustrating tan δ = 1, (b) applying preshear rates at 128 °C, and (c) induction time versus preshear rate.

### Linear Regression Analysis from Extrusion Experiments

The correlation between the processing, crystal structure, and properties of experimental results was investigated by using the multiple linear regression model and analysis of variance (ANOVA). These summary results are shown first, followed by a more detailed discussion of the relationship among the crystal structure, physical properties, and process variables. MATLAB software was employed to analyze the experimental results, fitting a multiple linear regression model with ANOVA. The estimated coefficients of the processing variables in the linear regression model, along with the corresponding *R*-squared values for fitting the linear regression model, are presented in Table 4. The processing variables with a statistically significant impact, as determined by a *p*-value less than 0.05, are indicated with an asterisk.

**Table 4:** Calculated Coefficients of Fitted Linear Regression Model

Factor	Crystallinity percentage (%)	Crystal size (A°)	Lamellar spacing (A°)	Orientation degree (%)	Modulus (MPa)	Tensile strain at break (%)	Tensile stress at break (MPa)	Permeation rate (cc·mil/m^2^/day)
**Intercept**	44.63*	140.8*	296.63*	44.55*	585.47	114.68*	39.946*	3924.4*
**T-Roll**	0.069*	0.081*	0.213	0.022*	2.463	–0.028	0.168*	–5.853
**Draw ratio**	–0.098	0.203	–3.017	–0.442*	98.361*	–4.6*	1.75*	43.792
**Flow** **rate**	0.873*	0.251	–0.894	0.104	40.041*	–3.23*	1.036*	42.099
**Shear** **rate**	0.011	–0.066	0.716*	0.003	1.185	–0.148	–0.078	–7.229
**Sonication**	–0.228	–2.32*	8.148	–0.552	9.353	–1.576	0.723	–132.95
**Anvil-Shear** **rate**	0.005	0.024	0.391*	0.008	–0.207	0.03	0.12	–7.124*
**Sonication-Shear** **rate**	0.004	0.02	–0.114	–0.002	–0.418	0.112*	0.003	2.593
**R** ^**2**^ **Values**	0.89	0.71	0.962	0.71	0.875	0.898	0.788	0.828

Regression analysis revealed that several processing parameters significantly influence the structural and mechanical behavior of HDPE. Crystallinity was most strongly affected by the flow rate, which showed a statistically significant positive correlation, indicating that increased flow promotes chain alignment and crystalline domain formation. T-Roll also made a positive contribution, albeit to a lesser extent. Crystal size was moderately influenced by the draw ratio and flow rate, while sonication had a significant negative effect, suggesting disruption of lamellar growth. Lamellar spacing was highly responsive to the shear rate and anvil-shear rate, both of which promoted wider spacing through flow-induced ordering. Orientation degree was slightly improved by T-Roll. Mechanical properties exhibited a strong dependence on the draw ratio and flow rate. The modulus increased significantly with both, indicating enhanced stiffness due to improved chain packing and crystallinity. However, the tensile strain at break decreased under the same conditions, suggesting reduced ductility. In contrast, tensile stress at break improved with draw ratio and flow rate, consistent with an increased molecular orientation. Permeation rate was most dramatically affected by sonication, which significantly reduced permeability—possibly due to densification or the formation of rigid amorphous fraction—while draw ratio and flow rate increased it, potentially by inducing micro-voids or orientation pathways. Overall, the model demonstrated excellent predictive power for lamellar spacing (*R* ^2^ = 0.962) as shown in Figure S4 of the Supporting Information, modulus (*R* ^2^ = 0.875), and crystallinity (*R* ^2^ = 0.89), confirming that processing parameters play a critical role in tuning HDPE’s structure–property relationships.

### Crystal Structure-Process Correlation

The impact of processing parameters as well as the ultrasonic field on HDPE’s crystallinity and crystal structure was analyzed using WAXS and SAXS techniques as summarized by the linear regression data and shown in Figure 5.

**Figure 5:**
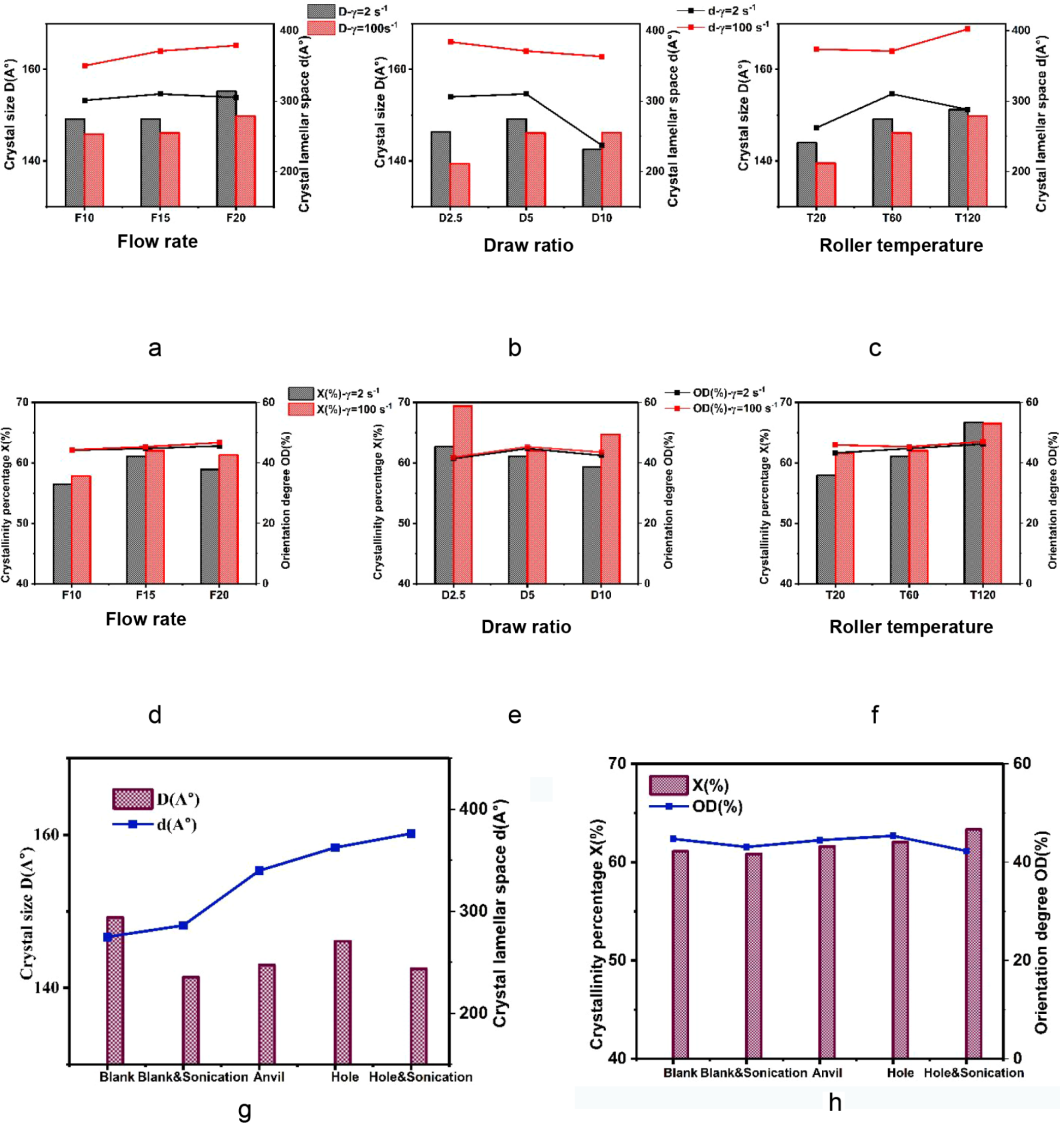
Dominant lamellar spacing (*d*) is represented by lines and crystal size (*D*) by bars; black and red indicate low and high shear rates, respectively, across varying (a–c) processing parameters and (g) ultrasonic plate types with an*d w*ithout sonication. Crystallinity percentage (*X*%) is shown in bars and orientation degree (OD%) in lines, again using black for low and red for high shear rates under different (d–f) processing conditions and (h) ultrasonic plate types with and without sonication.

The size of the lamellar spacing (*d*) is presented under various processing conditions in Figure 5a–c and under the influence of the ultrasonic field applied through different sonication plate geometries in Figure 5g in a line format. The results show a significant positive impact of the shear rate, including ultrasonically induced shear rates, on *d*. This observation aligns with the mechanisms of chain orientation and strain-induced rearrangement of crystalline lamellae that occur during drawing and cooling in cast film extrusion. The combined influence of shear and extensional stresses promotes the formation of more extended crystalline regions, thereby resulting in an increase in lamellar spacing.^43^ Furthermore, applying ultrasonic vibrations through various plate designs (rectangular opening, anvil, and hole) results in a slightly higher lamellar spacing due to the effect of the added ultrasonic energy on the polymer chains’ dynamics and relaxation.

The crystallite size (*D*) determined from the breadth of the peak at the 110 plane was slightly reduced under the influence of the shear rate and sonication as shown in Figure 5g in bar format. This reduction can be attributed to the generation of an inhomogeneous temperature profile across the melt in the presence of the ultrasonic field.^44^ Specifically, the ultrasonic dissipation energy increases the melt temperature and chain entropy, which leads to limited growth rate of the spherulites. Consequently, the oriented chains subjected to extensional stress form more nuclei with smaller crystal sizes. This aligns with the previous demonstrations that the stretching of polymer chains alters crystal morphologies and also the orientation of polymer chains within the amorphous phase.^45^ Conversely, an increase in roller temperature as shown in Figure 5c countered this effect due to delayed nucleation from the less severe undercooling.

The average crystallinity, *X* (%) and the orientation degree, OD (%) are depicted as a function of the processing variables in Figure 5d–f and versus shear rate with and without the ultrasonic field in Figure 5h in bar and line format, respectively. The results indicate that the percent crystallinity slightly increases with higher roller temperatures and flow rates, likely due to a longer overall cooling time. However, the overall percent crystallinity averages 60% across all conditions, which is attributed to the molecular weights of HDPE. Higher molecular weights result in a lower crystallinity percentage but higher mechanical toughness.^46^ The orientation degree, OD (%), under various processing conditions does not show a significant change. The regression analysis shows a slight negative effect of sonication on percent crystallinity. This behavior could be due to the viscous shear heating that causes an increase in the entropy energy of the polymer chain, potentially extending the crystallization induction time and slowing both nucleation and growth in the melt.^47^

### Physical Properties-Process Correlation

The evolution of crystal structure during cast film extrusion significantly impacts the mechanical properties of HDPE. The results of tensile tests are shown versus processing conditions in Figure 6 a–f, with the influence of sonication illustrated in Figure 6g.

**Figure 6:**
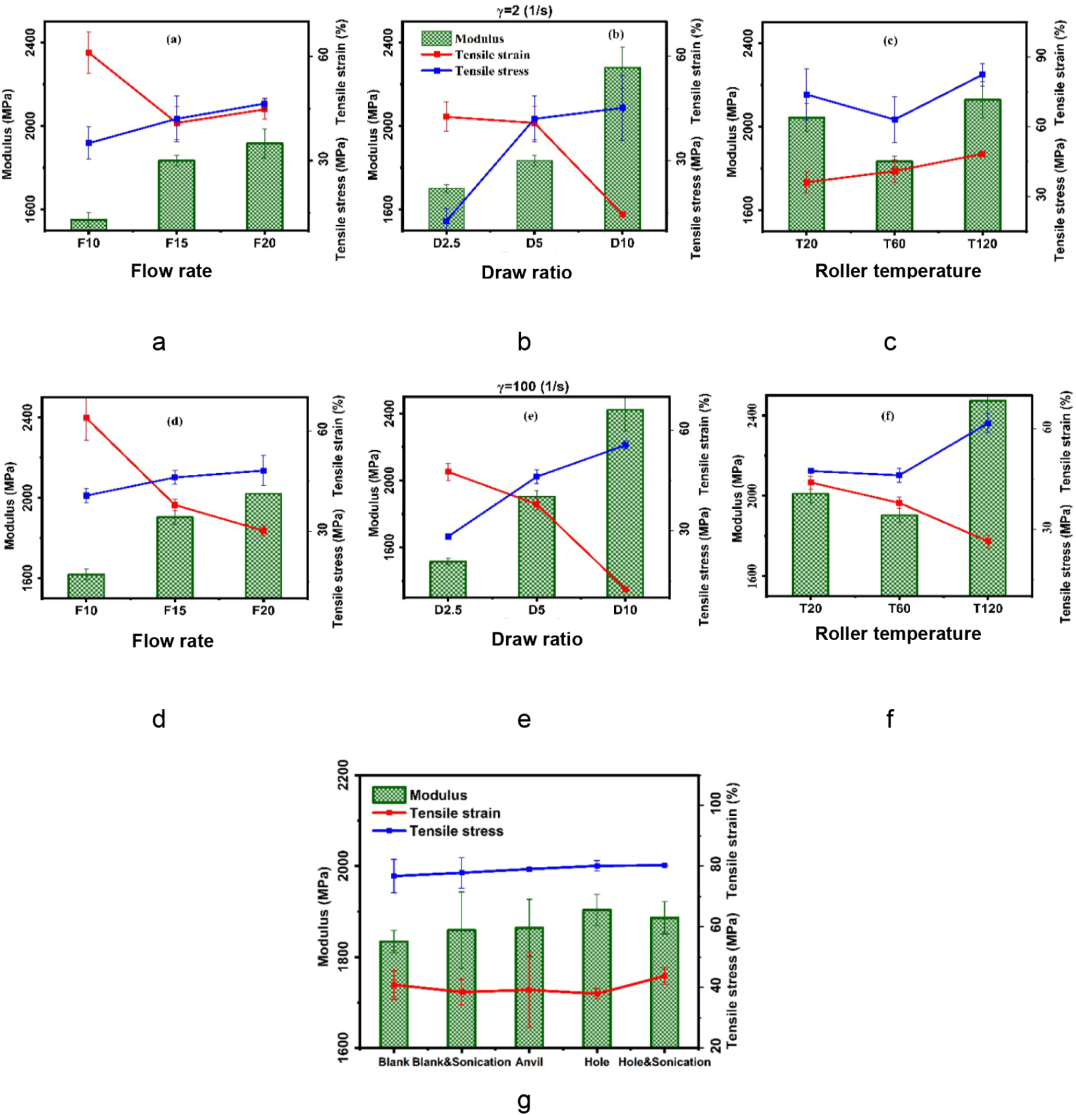
Mechanical properties (a–f) under various processing conditions and shear rates as well as (g) ultrasonic plate types with and without sonication.

HDPE shows a higher modulus and lower tensile strain at break as shear rate increases. This is primarily attributed to the enhanced crystallinity and the evolving crystal structure induced by cast film extrusion processing.^48^ Statistical modeling shows that shear rate reduces tensile strain at break but increases modulus due to changes in crystallinity and the formation of structurally constrained regions between crystalline domains as lamellar spacing increases. Lamellar spacing significantly impacts tensile strain at break and toughness by affecting rigid amorphous fraction formation through the polymer chain orientation. The application of shear through the sonication plate with holes contributes to increased toughness by decreasing the crystal size and expanding lamellar spacing within intercrystallite linkages, thereby enhancing stress transfer efficiency. This finding is consistent with established research indicating that greater lamellar spacing improves the polymer’s capability to dissipate stress due to the presence of tie molecules.^49^

The influence of processing on the barrier properties was assessed via the oxygen permeation rate (OTR), with results presented in Figure 7a–d. The average OTR reveals a reduction in oxygen transmission both with increasing shear rate and in the presence of an ultrasonic field. The enhanced barrier performance is attributed to the formation of a rigid amorphous fraction within the crystal domains. The higher lamellar spacing results in more chain alignment under higher shear in addition to increasing the crystallinity percentage. This result is consistent with prior findings that polyethylene with densified amorphous regions has enhanced barrier properties compared to pristine films.^49^ In addition to the quantitative crystal parameters discussed previously, the structure quality was evaluated using WAXS as depicted in Figure 7e, by comparing scattering intensity with respect to scattering vector q for the minimum and maximum OTR. The diffraction pattern displayed a third peak associated with the (010) crystal plane, which indicates the presence of a monoclinic phase alongside the prevailing orthorhombic phase. This monoclinic phase peak was most apparent for samples processed at lower flow rates using the hole plate (F10@*γ* = 100 s^–1^), resulting in the lowest OTR. This observation corresponds to shear-induced crystallization, as the monoclinic phase forms when stress exceeds the yield point. While this phase typically represents less than 10% of the overall polymer crystalline content, its formation is a marker of the development of an oriented structure within both amorphous and crystalline regions.^50^

**Figure 7:**
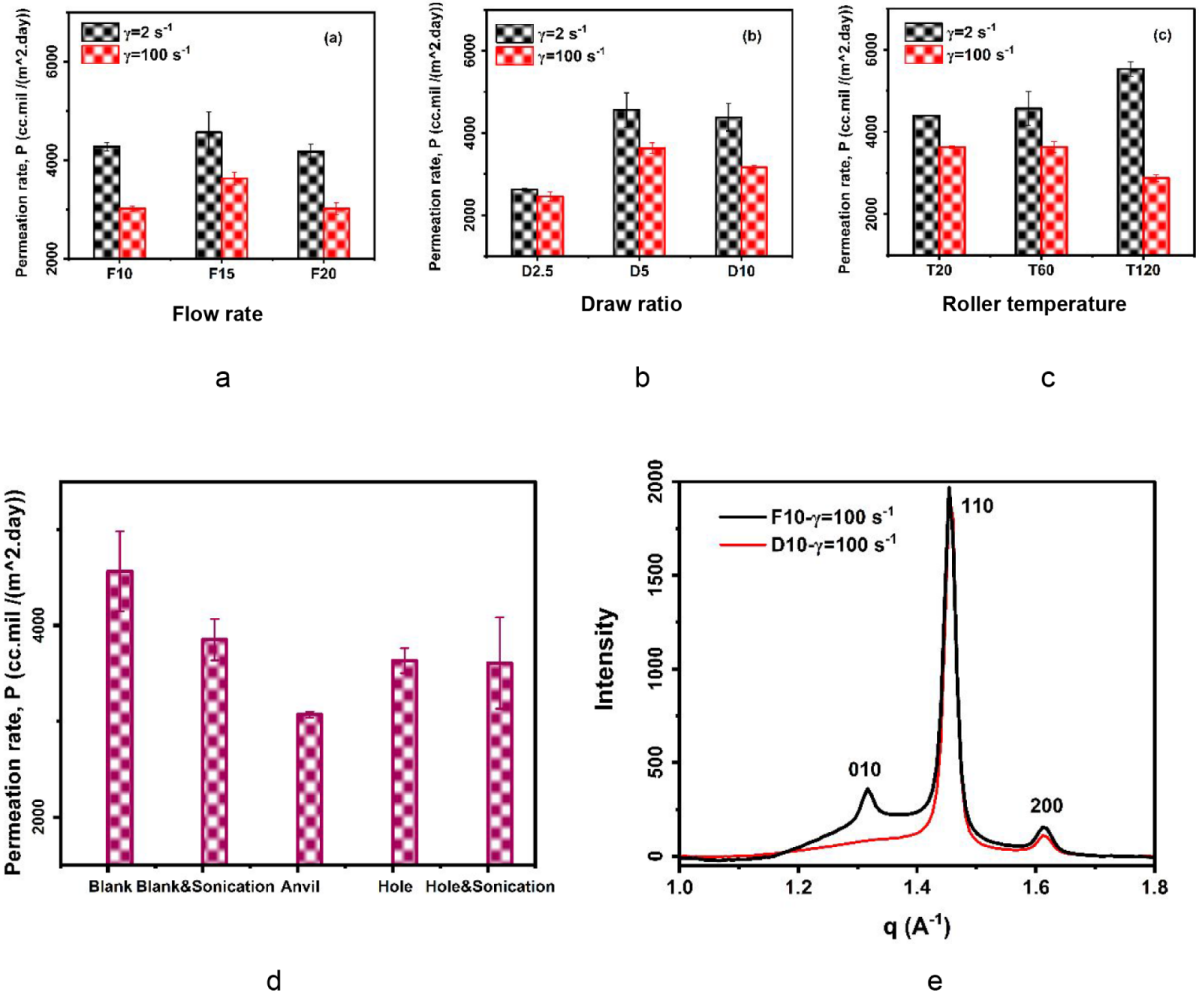
Oxygen permeation rate under (a–c) various processing conditions and shear rates, (d) ultrasonic plate types with and without sonication, and (e) normalized wide-angle scattering intensity vs *q* vector for low (F10@g = 100 s^–1^) and high (D10@g = 100 s^–1^) permeation rates.

The monoclinic phase appears to improve HDPE’s oxygen barrier properties. OTR is especially low with ultrasonic-assisted processing using the anvil pattern, likely due to changes in crystal structure and increased rigid amorphous fractions from the combined shear stress and ultrasound. Ultrasonic shearing delivers oscillatory stresses evenly throughout the polymer melt.^51^ Combining ultrasound with shear flow repeatedly stretches and relaxes polymer chains, encouraging structural reorganization. This is most evident in high shear melt regions, where ultrasound helps to create a refined, highly oriented crystalline structure.

## Conclusion

This research presents a comprehensive study investigating the correlation among the structure, processing conditions, and properties of HDPE using ultrasonic-assisted extrusion. To investigate the effects of conventional shear flow and ultrasonic shear on crystallization kinetics and crystal structure evolution, we designed an extrusion die with a sonication assembly featuring three distinct sonication plates was designed. The influence of process parameters, particularly the ultrasonic field, on the crystal structure of HDPE was analyzed to discover the influence on the mechanical and physical properties. The impact of the flow rate, draw ratio, cooling rate (roller temperature), and shear rate on crystallinity and crystal morphology was systematically examined using WAXS and SAXS.

Crystallization kinetics of HDPE was analyzed using the Ozawa model, confirming a nucleation-controlled growth mechanism under quiescent conditions. The effect of shear stress during cast film extrusion on the crystallization kinetics was evaluated by measuring the crystallization induction time after introducing different shear rates. Structural analysis revealed that shear rate (both conventional and ultrasonic-induced) had the most significant influence on lamellar spacing, ultimately dictating the mechanical and physical properties of the films. The behavior is due to the redistribution of rigid and soft amorphous fractions within the crystalline domains. HDPE exhibited a more brittle fracture and lower oxygen permeability due to the formation of a more rigid amorphous fraction. The structure-process-property relationship under ultrasonic-assisted extrusion revealed a negative impact on the crystal size. This result can be attributed to energy dissipation and a higher temperature of the extrudate at the die. However, the applied shear stresses resulted in increased lamellar spacing and enhanced polymer chain orientation in the amorphous phase, leading to distinct physical properties. The result of WAXS revealed the formation of monoclinic crystal structures under shear stress, leading to more brittle mechanical behavior and being associated with reduced oxygen permeability.

Although ultrasonic-assisted extrusion processing decreased the OTR of the neat polyethylene film, the resulting barrier properties are still higher than those of commercial high-barrier packaging. Consequently, this study serves as an important initial step toward enhancing the intrinsic barrier capabilities of polyolefins to achieve practical application standards. For future research and the refinement of crystallization kinetics, it is advisable to apply sonication immediately prior to the melt reaching its peak crystallization temperature. Given the challenges associated with extruding polymer melts at such low temperatures, an alternative strategy would be to locate the sonicator at the die lip, thereby maximizing its influence on the crystallization process. Additionally, when optimizing ultrasound-assisted crystallization, it is essential to consider the orientation of the ultrasonic field in relation to the direction of the melt flow. Aligning the ultrasound application with the flow direction can further develop the inherent shear stress within the system, resulting in enhanced molecular alignment, increased nucleation, improved lamellar orientation, greater crystallinity, and superior film properties.

## Supplementary Material



## Notes

Rheology testing protocol, regression modeling, DSC data, and X-ray diffraction curves (PDF)
