# Supplementary material for: Ultrasonic-Assisted Extrusion Processing for Enhancing Physical Properties of High-Density Polyethylene by Flow-Induced Crystallization
Source: ACS Appl Polym Mater. 2026 Jan 6;8(2):839–50. doi: 10.1021/acsapm.5c03508 (PMC12836317; doi:10.1021/acsapm.5c03508)
Supplement: Supplementary file 1 [file ap5c03508_si_001.pdf]

Ultrasonic-Assisted Extrusion Processing for Enhancing Physical Properties of High-density Polyethylene by Flow-Induced Crystallization

Mansoureh Jamalzadeh [a], David O. Kazmer [a], Patrick Casey [a],
E. Bryan Coughlin [b], Margaret J. Sobkowicz [a]*

a) Plastics Engineering Department, University of Massachusetts Lowell, MA 01854 USA

b) Polymer Science and Engineering Department, University of Massachusetts Amherst, MA 01004 USA

*Margaret_sobkowiczkline@uml.edu

Supporting Information

The rheology protocol for studying flow-induced crystallization using the parallel plate rheometer is presented in Figure S1.

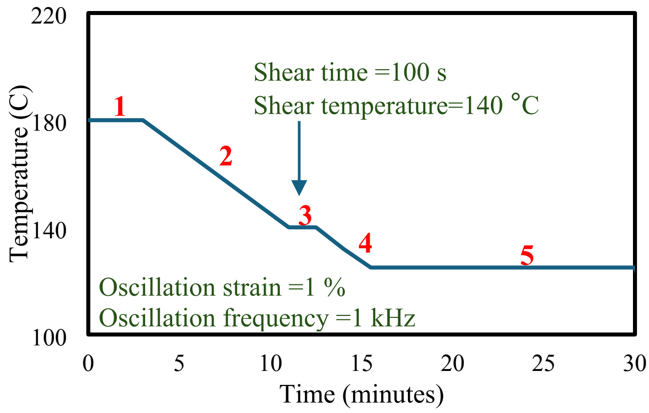

Figure S1: Rheology protocol for examining isothermal shear induced crystallization of HDPE

The crystallization peak and melting temperature of selected HDPE were shown in Figure S2.

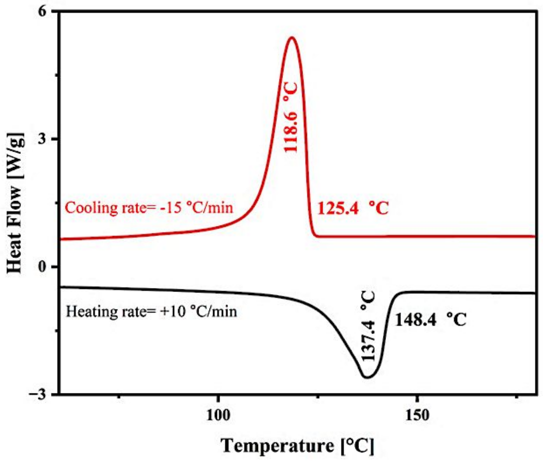

Figure S2: Differential scanning calorimetry showing the thermal transitions of HDPE

The zero shear viscosity of HDPE at 140°C in the low-frequency region is illustrated in Figure S3.

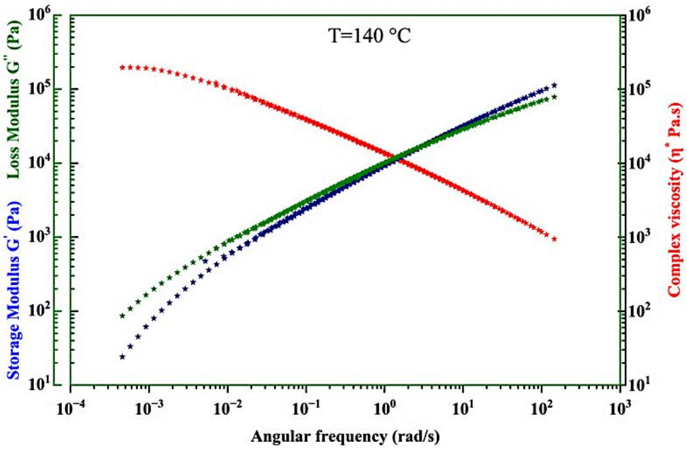

Figure S3: HDPE Master curve at T=140 °C

The fitted regression model for the lamellar spacing of HDPE crystals demonstrates a high coefficient of determination (R^2), as shown in Figure S4.

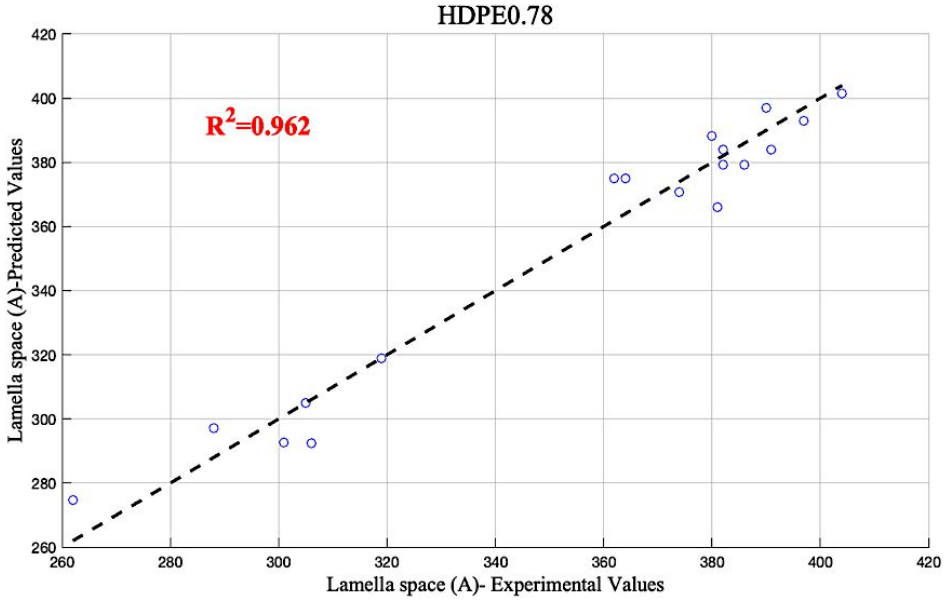

Figure S4: Linear regression model fitted to characterize the lamellar spacing

The X-ray scattering patterns obtained from WAXS measurements are shown in Figure S5 as a function of the processing parameters.

a b c

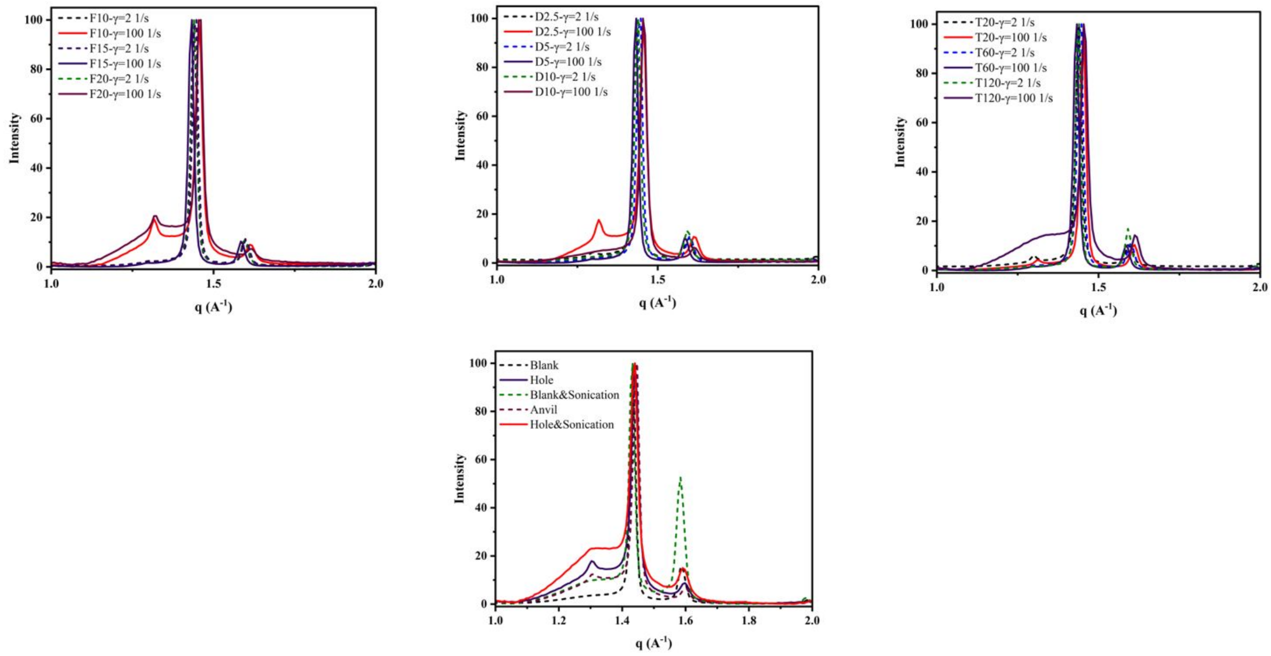

d

Figure S5: Normalized scattering intensity versus scattering vector q under, (a-c) various processing conditions and shear rates, (d) ultrasonic plate types with and without sonication
